# Social Isolation and Aging in Zambia: Examining the Possible Predictors

**DOI:** 10.1155/2012/537467

**Published:** 2012-08-08

**Authors:** Christopher Chabila Mapoma, Gift Masaiti

**Affiliations:** ^1^Department of Population Studies, School of Humanities and Social Sciences, University of Zambia, Box 32379, Lusaka, Zambia; ^2^Institute of Economics of Education and Management, Graduate School of Education, Huazhong University of Science & Technology, Wuhan 430074, China

## Abstract

This research paper examined social isolation and aging in Zambia by examining possible predictors. The paper produces evidence on risk factors likely to engender social isolation among the elderly population of Zambia. Snowball sampling was undertaken to select 690 adults aged 60 and over in communities as well as those living in homes for the aged. A structured questionnaire was used to solicit information from respondents. Results show that old people in Zambia experience forms of social isolation which exhibit themselves (but not limited to) through such factors as loss of appetite, stress, moody, hopeless, useless, unhappy, and lonely. On balance, however, the direction of association and the number of statistically significant findings suggest that associations between variables examined and risk factors associated with social isolation amongst older people in this analysis could explain the overall situation occuring currently in Zambia and probably other developing countries. In view of this, this study recommends that further work is needed to identify and explain details of factors of social isolation using techniques such as focus group discussions as well as in-depth interviews with key informants. Such approaches may even help to explain why, for example, sex seems not to be significant in determining indicators of social isolation.

## 1. Introduction

Studies on ageing, particularly risk factors that may engender social isolation amongst the elderly population in Zambia, are almost nonexistent currently. In fact, very little has been written on ageing in Zambia in general. While it is true that some studies have been conducted [1–5] by Cliggert [6] none of such studies have based their arguments on possible risk factors likely to foster social isolation among the elderly. Further, not only have these studies been devoid of details around the subject itself, but have also tended to be speculative and largely been based on little or no empirical evidence derived from any extensive systematic investigation. In view of this lacuna, therefore, this paper aimed at producing evidence on risk factors likely to engender social isolation among the elderly population of Zambia.

## 2. Social Isolation and Associated Risks

Social isolation has been variously defined in the literature [7]. For example, Day [8] defined it as “the absence of satisfying relationships and a low level of involvement in community life” (page 7). Gardner et al. [9] considered participants in their study to be socially isolated if they were experiencing a combination of factors that included low levels of social participation and levels of social activity that the older person perceived as inadequate. Cattan and White [10] and Haddad and Gillepie [11] define social isolation as the objective measure of having minimal interaction with others, and emotional isolation (or loneliness) as the subjective feeling of dissatisfaction with having a low number of social contacts.

On the other hand, there are many risk factors that can lead to social isolation. Those identified in the literature include loss—for example, loss of social capital to look after themselves adequately, loss of respect, and so on—(in its many forms), poor health, mental illness, care giving, geographic location, communication difficulties, being male and single, and transport difficulties [9, 11–14] It should be mentioned also that, while loneliness is a subjective feeling of dissatisfaction [7], it was, however, considered a risk factor for social isolation in our study. It has been argued that social isolation and risks associated with it are to some extent a function of some of the factors identified in this paper. They include such experiences as loss of appetite, being stressed, moody, feeling hopeless, useless, and being unhappy. All these factors may contribute significantly or may be prompted by experiences related to social isolation or may actually indicate the presence of social isolation in general. For example, it is possible for an individual to be unhappy or feel useless because they were experiencing low levels of social participation and activity in society.

## 3. Objectives and Methods

The main objective of this study was to investigate the likely effects of social-demographic attributes such as age, sex, residence, marital status, and education on social isolation risk factors. The main aspect this article addressed was the extent to which social-demographic variables impact risk factors associated with social isolation among the elderly in Zambia.

The research design for this study was quantitative; data was collected through a structured questionnaire from elderly people aged 60 and above living in communities and homes for the aged. Snowball sampling, [15, 16] was used to select these respondents. Samples were drawn from five towns, namely, Sesheke, Livingstone, Lusaka, Ndola, and Mufulira. These towns were selected because they have homes for the aged. The homes for aged included Kandiana, Maramba, Matero, Mitanda, and Chibolya. A total of 690 respondents were interviewed using the structured questionnaire mentioned earlier. This sample size was representative enough and using Fraenkel and Wallen's [17] argument; a minimum of 100 is recommended for descriptive studies, while for correlational studies, a minimum of 500 is recommended.

## 4. Measures and Analysis

The current analysis focuses on seven indictors of social isolation. Specifically, respondents were asked whether they experience any or all of the following: loss of appetite, stress, being moody, hopeless, useless, unhappy, and loneliness. A “yes” response meant indication of isolation or something related to it and a “no” meant the opposite, that is, the respondent was not experiencing social isolation or at least not at the time of data collection.

On descriptive outputs, such as the total sample and related percentages, simple cross tabulations were performed to present frequencies by sex, age, residence, education, and marital status. For multivariate analysis, binary logistic regression was utilized but with the same correlates categorized for descriptive analyses.

## 5. Results

Data in Table 1 shows that the sample contained more females than males; the mean age for the population was about 72 years, with more respondents from the urban settings than rural areas. Further, the table also shows that majority of respondents were widowed, with females exhibiting a higher percentage compared to males. In the same way, more males than females reported being married. In fact, this is a reflection of the norm in most African societies. Overall also, primary education attainment is more prominent (52%) compared to higher education (15.8%) and those reporting never having attended school (32%), respectively.

Table 2 shows descriptive statistics of reported social isolation indicators. The table shows cross-tabulations between primary predictors and social isolation indicators.

It is evident from Table 2 that over half of respondents (52%) were actually lonely, with another half (50%) indicating that they were stressed up; over one in four (30%) and at least 2 in 5 reported that they feel hopeless and unhappy, respectively. Although proportions of other indicators were not as high as these four (loneliness, stress, hopeless, and unhappy), the fact that they were in referent here shows both their impact on respondent's lives and their ability to influence older people presumably in the negative.

About 1 in 5 (20%) reported that they often lose appetite; while it is true that this is not strictly a problem of the elderly, sometimes older people are said to be problematic in what they prefer to eat; this therefore affects how they view their eating habits.

Over three-quarters indicated that they felt moody (28%); however, this may not necessarily be caused by their age; such feelings are sometimes exacerbated by the manner society treats the elderly in general—for example, being reminded that one is old and therefore worthless or unwanted could be one of the reasons. In addition, at least 1 in 4 (27%) respondents reported that they feel useless. All these aspects are part of the challenging social, physical, and economic environments old people are faced with.

Social isolation was also visible through other forms which may be explained by understanding social networks through contacts as it were. Loneliness, for example, is both a “social contact problem” as much as being a self-esteem indicator. In this example, results suggest that feeling lonely depends also on one's age, and loneliness increases with age. As it were, over 3 in 5 (67%) of those aged 80+ reported that they were lonely; this contrasts those aged between 60 and 79. Specifically, those aged between 70 and 79 were also more likely to report being lonely compared to those aged between 60 and 69; in any case although they also experience a great deal of loneliness (70–79 age group), it is not as pronounced as that reported by those aged 80 and above. Similarly, over half of female respondents (55%) reported that they were lonely. This of course is higher than what males reported (47%). The difference observed on loneliness between males and females can be understood presumptively. Females in these age groups are more likely to be either staying alone or widowed and as a result feel lonelier than males [18]. In many instances, men, young or old, remarry in case of marriage cessation; they are therefore “saved” from being lonely [19].

Widowhood, never having been married before and having no education, influences one's exhibit of social isolation. This is evident in Table 2 and almost consistent with the social isolation indicators being examined herewith.

Residence is pivotal in determining loneliness as well. It seems that older people in rural areas are more at risk of being lonely than those in urban areas. This is so because close to three-quarters (73%) of respondents in rural areas reported being lonely compared to about 47% in urban areas. Understandably, more than 40 percent of respondents reporting to be lonely in urban areas are also a significant lot, indicating the extent to which elderly people are lonely despite residing in an area which under normal circumstances should have accessible social amenities including some form of entertainment as it were.

Other measures of social isolation do not depart extensively from what has been observed above. These measures are also affected by age, sex, and residence disproportionately. For example, over half of respondents aged 80+ (54%) reported that they were unhappy; close to half of those reporting being unhappy were women. Further, majority of respondents reporting being unhappy were from rural areas. The oldest old (80+), mostly women and respondents residing in rural areas, reported that they felt more useless, more hopeless, and moody compared to those aged from 60 to 79, residing in urban areas and being male respondents, respectively.

Being stressful was another condition reported by respondents in our study. Stress is synonymous with old age [20]. However, it is also highly experienced by young adults in demanding midcareers and by young mothers as they raise small but problematic children; it is also common in mothers who have children with specific functional problems, for example, brain problems, paralysis, disability, and so forth [21]. As early as the early 1990s, studies have been conducted to measure stress-related problems mostly in developed countries but also in some fast developing countries like the Asian Tigers. In China, for example, a study was conducted to investigate the relationship among stress, social support, and depressive symptoms in adults. It was discovered that stress is mostly an end in itself and basically brought about by other significant factors, especially social support [22]. Other causal factors include financial strain and emotional support. However, these factors may erode each other depending on the intensity, and it has been documented that financial support is not necessarily a remedy for such emotional strain [21].

Results herewith indicate that as many as half of respondents (50%) reported that they were stressed up. Stress has many causes. In explaining some of them, Phiri [4] states that elderly persons are exposed to various types of stress-causing factors ranging from adjusting to retirement and reduced income, deaths of spouses or friends, loss of status or living condition as a result of retirement, illness, hormonal changes, failing faculties, and ageism. Possibly, these reasons may also explain why majority of respondents in this study reported being stressed; in any case, this aspect requires further research or investigation.

The most stressed age category is the 70–79; close to 3 in five (58%) of people in this age group reported that they were stressed. Further, females and those residing in communities reported being more stressed compared to men and those residing in homes for the aged. Females seem more stressed than men due to several reasons but pertinent amongst them are their major roles as wives as well as in most cases being breadwinners. It has been documented that females are breadwinners for families whose livelihoods are dwindling as a result of limited external support. If, for example, she is keeping orphaned children, most likely, she may experience stress due to challenges such as finding means to feed the children; if some of the children are in school, she has to find means of ensuring that school requirements are in place. While it is true that men are also affected, the trend is more pronounced in women.

On the other hand, rural respondents, the never married and those with no education, reported being more stressed compared to other sociodemographic variables in the table. While being stressed up is not subject to an area, it is true in this data that older people in rural areas seem to be more stressed than those in urban areas. It has, however, been suggested that urban life is more demanding and more stressful compared to rural life. Nonetheless, this depends largely on a number of varying factors which may not be universally true. It is possible, for example, that those majority respondents in urban areas may be kept or at least stay with a relative or close friend and therefore are able to share responsibilities, or they may have a pension, and so forth. Given such a scenario, it may necessarily offset negative stress experiences which privilege people in rural areas may not have. Similarly, rural elderly could mostly be alone and their day to day life experiences maybe more challenging and demanding and subsequently affect how they respond to stressful situations.

## 6. Predictors of Social Isolation

Table 3 presents multivariate results examining some common social and demographic predictors of social isolation. It is hypothesized that age and sex influence chances of experiencing social isolation through the afore-mentioned indicators. Further, it is also assumed that marital status, education, and residence determine one's experiences of social isolation.

In exception of “being moody,” results in Table 3 show that there are statistically significant relationships between the named primary predictors in the logistics regressions model and social isolation indicators. However, note that one's sex has no true bearing on one's experiences of social isolation; this is so because the odds ratios in the equation were not statistically significant.

Specifically, one' age has a bearing on respondents' report of loss of appetite for example. However, one's age does not, in this case, determine whether one would be moody, hopeless, and useless, respectively. Note that age has a weak appeal to one's being unhappy as well as being lonely. On the other hand, marital status influences one's loss of appetite, being unhappy, and being lonely; it does also have an influence on stress, although much weaker comparatively.

Education is also significant in determining one's reporting on feeling useless as well as being lonely; it also has a weak bearing on reported hopelessness as well. Similarly, one's residence (urban or rural) strongly determines one's feeling of hopelessness, useless, unhappy, and being lonely. In fact, residence—whether one stays in urban or rural areas—affects more of the chosen social isolation indicators compared to other predictors in the equation.

## 7. Discussion

Zambia, like many developing countries, is prone to the ageing factor, and measures aimed at studying this phenomenon extensively need particular attention. The current analysis employed recently collected data, although at a minimum to assess likely effects of sociodemographic variables such as sex, age, marital status, residence, and education on risk factors or indicators associated with social isolation.

As people age, they are also more likely to experience social isolation which may exhibit itself (but not limited to) with such factors as loss of appetite for no apparent reason, being stressed, moody, hopeless, useless, unhappy, and loneliness. Being socially isolated or feeling that one is socially isolated due to one's age inevitably affects how they age and most likely affects active aging in general. As such, social isolation indicators are not only indicative of inactive aging as it were but also a challenge to the existing social and physical needs of older people. On balance, however, the direction of association and the number of statistically significant findings suggest that associations between variables examined and risk factors associated with social isolation amongst older people in this analysis could explain the situation occuring currently in Zambian and probably other developing countries.

Results in this analysis show that while descriptive indicators suggest that one's sex plays a significant role in determining risks associated with social isolation amongst the elderly, logistic regression results do not seem to support this assertion; in other words, being male or female has little or no bearing on risks associated with social isolation. This to some extent defies what has been found in the literature and what is the norm—usually, while females generally live longer than males, they also suffer the most not just physically but socially as well [23]. Age on the other hand is a significant determinant, and social isolation increases with one's age. In addition, marital status, especially if one reported never married or widowed, plays a significant role in determining social isolation. Married elderly people are more resilient to risks of social isolation and seem to be oblivious to such environments. This also is well established in the literature [24]. Education and residence are significant determinants of social isolation; elderly people with no education and residents of rural areas are more likely to experience social isolation in general.

## 8. Conclusion

Many lessons can be drawn from these findings, but the main contribution is the reporting on a social situation about which very little is known. Studies on aging in Zambia are still in their infancy and while it is true that the current analysis is based on what may be termed as a relatively small sample size, these results may inevitably depict the situation as it occurs on the ground. In view of this, further work is needed to identify and explain details of factors of social isolation using techniques such as focus group discussions as well as in-depth interviews with key informants. Such approaches may even help to explain why, for example, sex seems to be insignificant in determining indicators of social isolation as it were.

## Figures and Tables

**Table 1:** Description of sample of elderly Zambians (60 years and above).

	Total population	Males	Females
*N*	690	284	406
Mean Age	72.1	73.7	71

% Residence			
Urban	81.7	86.2	13.8
Rural	18.3	78.5	21.5

% Marital status			
Never	3.77	4.23	3.45
Married	35.8	56.34	21.43
Divorced/separated	11.45	13.03	10.34
Widowed	48.99	26.41	64.78

% Education			
Never	32.17	18.66	41.63
Primary	52.03	55.63	49.51
Higher	15.8	25.7	8.87

**Table 2:** Percent reporting indicators of social isolation by sex, age, residence, marital status, and education.

	*N*	Appetite loss	Stress	Moody	Hopeless	Useless	Unhappy	Lonely
Population	690	20.7	50.6	28.6	30.0	27.3	44.8	52.3

Males	284	21.1	45.8	27.5	27.1	23.9	40.1	47.5
Females	406	20.4	53.9	29.3	32.0	29.6	48.0	55.6

60–69	299	15.4	44.5	26.1	27.1	22.7	39.8	43.1
70–79	253	24.1	58.1	30.8	31.2	26.9	45.5	54.9
80+	138	26.1	50.0	29.7	34.1	37.7	54.4	67.4

Urban	564	19.3	48.9	29.1	26.4	23.8	41.5	47.7
Rural	126	27.0	57.9	26.2	46.0	42.9	59.5	73.0

Never married	26	30.8	53.9	46.2	42.3	50.0	57.7	69.2
Married	247	13.8	43.3	23.9	23.5	17.8	34.8	35.6
Divorced/separated	79	26.6	48.1	25.3	36.7	31.7	43.0	48.1
Widowed	338	23.7	56.2	31.4	32.3	31.4	51.5	64.2

No school	222	20.3	56.3	32.0	35.6	35.6	50.5	61.3
Primary	359	22.3	49.0	26.7	29.0	26.5	44.0	52.7
Higher	109	16.5	44.0	27.5	22.0	12.8	35.8	33.0

**Table 3:** Logistics regression results predicting indicators of social isolation.

	Appetite loss	Stress	Moody	Hopeless	Useless	Unhappy	Lonely
Age	0.744^∗∗^	0.886	0.908	0.952	0.828	0.816^+^	0.729^+^
Sex	1.149	0.835	0.948	0.971	0.978	0.867	1.072
Marital status	0.812^∗∗^	0.868^+^	0.948	0.934	0.910	0.838^∗∗^	0.682^∗∗∗^
Education	0.924	1.134	1.089	1.301^+^	1.608^∗^	1.137	1.399^∗∗^
Residence	0.677^+^	0.742	1.204	0.435^∗∗∗^	0.443^∗∗∗^	0.517^∗^	0.351^∗∗∗^

*P < 0.01; **P < 0.05; ***P < 0.0001; ^+^ *P* < 0.10.

## References

[1] Colson E, Thayer S (1975). *New Economic Relationships Between Children and Parents*.

[2] Kamwengo M (2001). *Ageing and the Elderly in Zambia: Perspectives and Issues*.

[3] Kamwengo M (2004). *Growing Old in Zambia: Old and New Perspectives*.

[4] Phiri N (2004). *A phenomenological study of ageing amongst the older persons in Zambia [Ph.D. dissertation]*.

[5] Toner A (2005). *Grains from Grass: Aging, Gender and Famine in Rural Africa*.

[6] Cliggertt L, Parkin D (2005). *My Mother's Keeper: Changing Family Support Systems For the Elderly in Eastern Africa, Edited By*.

[7] Duflo E (2003). Grandmothers and granddaughters: old-age pensions and intrahousehold allocation in South Africa. *World Bank Economic Review*.

[8] Day A Opening address.

[9] Gardner I, Brook E, Ozanne E, Kendig H (1998). *Improving Social Networks: A Research Report*.

[10] Cattan M, White M Health promotion interventions targeting social isolation and loneliness.

[11] Haddad L, Gillespie S (2001). Effective food and nutrition policy responses to HIV/AIDS: what we know and what we need to know. *Journal of International Development*.

[12] Brennan PF, Moore SM, Smyth KA (1995). The effects of a special computer network on caregivers of persons with Alzheimer’s disease. *Nursing Research*.

[13] Edelbrock D, Buys L, Creasey H, Broe GA (2001). Social support, social networks and social isolation, the Sydney older persons study. *Australasian Journal on Ageing*.

[14] Havens B, Roos NP (1991). Predictors of successful ageing: a twelve year study of Manitoba elderly. *American Journal of Public Health*.

[15] Goodman M, Schular S, Ross JL (1983). Social and economic forces affecting Gwembe Valley and the line of rail. *Town and Country in Central and Southern Africa*.

[16] Case A, Deaton A (1998). Large cash transfers to the elderly in South Africa. *Economic Journal*.

[17] Fraenkel JR, Wallen NE (2003). *How to Design and Evaluate Research in Education*.

[18] Knodel J, Chanpen S, Obiero W (1999). Do small families jeopardize old-age security? Evidence from Thailand. *Bold*.

[19] United Nations Population Fund (2008). *World Population Status Report*.

[20] Gironda E, Lubben M, Coleman D (1996). Population ageing in Europe. *Europe’s Population in the 1990s*.

[21] Ogg J (2005). Social exclusion and insecurity among older Europeans: the influence of welfare regimes. *Ageing & Society*.

[22] Knodel J, Chanpen S (2007). Rural parents with urban children: social and economic implications of migration for the rural elderly in Thailand. *Population, Space and Place*.

[23] WHO/IASO/IOTF (2000/2003) *Asia-Pacific Perspective: Redefining Obesity and its Treatment*.

[24] Sugiswawa P, Giudici C (1994). *Demographic Implications of Social Exclusion in Central and Eastern Europe*.

